# Neonatal Conjugated Hyperbilirubinemia: Clinical Profile, Etiology, and Predictors of Adverse Outcomes in a NICU of a Tertiary Care Center

**DOI:** 10.7759/cureus.107724

**Published:** 2026-04-26

**Authors:** KNV Ramya, Sanjeev Kumar Verma, Manisha Verma, Prerna Priyadarshini, Shakal N Singh, Shalini Avasthi, Suruchi Shukla

**Affiliations:** 1 Pediatrics, King George's Medical University, Lucknow, IND; 2 Pediatrics, Hind Institute of Medical Sciences, Lucknow, IND; 3 Microbiology, King George's Medical College, Lucknow, IND

**Keywords:** biliary atresia, conjugated hyperbilirubinemia, etiological spectrum, neonatal cholestasis, neonatal liver disease

## Abstract

Background and objectives: Neonatal conjugated hyperbilirubinemia is a pathological condition reflecting cholestasis and hepatobiliary dysfunction, associated with significant morbidity and mortality if not detected early. Early recognition is critical, especially for surgically correctable or treatable causes such as biliary atresia and inherited metabolic liver diseases. Limited region-specific data exist from Northern India. This study aimed to characterize the clinical and biochemical profile, delineate the etiological spectrum, and identify predictors of adverse outcomes among neonates admitted to a tertiary care neonatal intensive care unit (NICU).

Methodology: A prospective observational study was conducted over one year in the NICU of King George’s Medical University (KGMU), Lucknow. Neonates with conjugated hyperbilirubinemia (direct bilirubin >1 mg/dL if total bilirubin ≤5 mg/dL or >20% if total bilirubin >5 mg/dL) were consecutively enrolled. Clinical, laboratory, microbiological, and imaging evaluations were performed. Outcomes were classified as good (discharged) or adverse (expired/leave against medical advice), and multivariate logistic regression identified independent predictors of poor prognosis.

Results: Seventy neonates were enrolled. Sepsis was the predominant etiology (41.4%), followed by intestinal pathology (27.1%). Multisystem involvement was frequent, with respiratory distress (91.4%), hepatomegaly (60%), encephalopathy (52.9%), and lactic acidosis (60%). Overall mortality was 50%, with 31.4% discharged. Adverse outcomes were significantly associated with elevated bilirubin, coagulopathy, hypoalbuminemia, and anemia. Independent predictors included neonatal acute liver failure (adjusted OR (aOR): 5.40; 95% CI: 2.10-11.20; p=0.0001), multiple organ dysfunction (aOR: 4.2; 95% CI:1.67-10.58; p= 0.003), metabolic disorders (aOR: 3.1; 95% CI:1.14-8.42; p=0.027), intestinal pathology (aOR:2.9; 95% CI:1.21-6.87; p=0.015), culture-positive sepsis (aOR: 2.6; 95% CI:1.14-5.98; p=0.02 ), and prolonged total parenteral nutrition (aOR: 2.3; 95% CI: 1.01-5.12; p=0.047 ).

Conclusion: Neonatal conjugated hyperbilirubinemia in this tertiary NICU cohort was predominantly driven by sepsis and intestinal pathology, with high mortality. Early, structured evaluation and targeted management of high-risk neonates may improve outcomes.

## Introduction

Neonatal jaundice is one of the most frequently encountered clinical conditions in the early neonatal period, affecting approximately 70-80% of term neonates and an even higher proportion of preterm infants [1,2]. It is characterized by yellow discoloration of the skin, sclera, and mucous membranes resulting from elevated total serum bilirubin levels [3]. In most cases, jaundice is physiological and transient, predominantly reflecting unconjugated (indirect) hyperbilirubinemia due to hepatic immaturity, increased bilirubin production, and enhanced enterohepatic circulation. In contrast, conjugated (direct) hyperbilirubinemia is always pathological and represents a key biochemical hallmark of neonatal cholestasis, necessitating urgent and systematic evaluation [4].

Neonatal cholestasis is defined as impaired bile formation and/or bile flow, leading to intrahepatic accumulation of bile constituents, particularly conjugated bilirubin and bile acids [5]. It is typically diagnosed when serum conjugated bilirubin exceeds 1.0 mg/dL, indicating hepatobiliary dysfunction [6]. Unlike unconjugated hyperbilirubinemia, conjugated hyperbilirubinemia is never physiologic and mandates further investigation to identify underlying hepatocellular, metabolic, infectious, endocrine, or obstructive etiologies. The estimated incidence of neonatal cholestasis is approximately 1 in 2,500 live term births, although it remains under-recognized due to overlapping clinical features with benign neonatal jaundice and its relative rarity [7].

The etiological spectrum of neonatal cholestasis is broad and heterogeneous. Extrahepatic obstruction, particularly biliary atresia (BA), accounts for 25-40% of cases and represents a time-critical diagnosis, as early surgical intervention significantly improves native liver survival [8]. Intrahepatic causes include genetic and syndromic conditions such as progressive familial intrahepatic cholestasis and Alagille syndrome, inborn errors of metabolism (IEMs), congenital infections, bile acid synthesis defects, and endocrine disorders [9]. The advent of next-generation sequencing has refined diagnostic classification and facilitated the identification of rare monogenic disorders, reducing the proportion of cases previously labelled idiopathic [10]. Nevertheless, comprehensive etiological workup remains challenging in many resource-limited settings.

Premature and small-for-gestational-age neonates are particularly vulnerable to cholestasis due to immature hepatobiliary transport systems and increased exposure to secondary insults. Prolonged parenteral nutrition (PN), especially formulations containing soy-based lipid emulsions, is a well-established contributor, with PN-associated cholestasis reported in up to 20% of neonates receiving PN beyond two weeks [11]. Additional risk factors include delayed initiation of enteral feeds, intestinal failure, and neonatal sepsis [12]. Importantly, the incidence of BA and other congenital cholestatic disorders in preterm infants appears comparable to that in term neonates, underscoring the need for equivalent diagnostic vigilance across gestational ages [13].

Although neonatal hyperbilirubinemia is most commonly unconjugated, the identification of conjugated hyperbilirubinemia should prompt immediate and comprehensive evaluation, as delayed diagnosis, particularly in BA, is associated with poorer outcomes [2,8]. In low- and middle-income regions, including several parts of Northern India, delayed referrals, limited access to advanced diagnostic modalities, and lack of standardized screening protocols further impede timely recognition and intervention [14].

Despite its clinical significance, region-specific data on the clinical presentation, etiological spectrum, and outcome determinants of neonatal cholestasis remain limited in Northern India. Such data are essential to inform context-appropriate diagnostic algorithms and optimize early referral strategies. Therefore, the present study aims to comprehensively characterize the clinical and laboratory profile of neonates with conjugated hyperbilirubinemia admitted to the NICU of a tertiary care center in Northern India, and to identify etiological patterns and factors associated with adverse outcomes.

## Materials and methods

Study design and setting

This prospective observational study was conducted over a one-year period in the NICU of the Department of Pediatrics at KGMU, Lucknow, a tertiary care referral center in Northern India.

The sample size was calculated using the standard formula for calculating proportions, based on the expected frequency of the primary outcome in the study population. As data on neonatal cholestasis outcomes in NICU settings are limited, an anticipated outcome proportion of 50% was assumed to yield the maximum sample size. With a 95% confidence interval and 12% absolute precision, the minimum required sample size calculated was 67. This was rounded off to 70 to ensure adequate statistical power and to account for the potential attrition during the study period. This study was approved by the Institutional Ethics Committee of KGMU, Lucknow (Approval No. 2421/Ethics/2024; Ref. Code: XXVII-PGTSC-IIA/P16; IEC Registration No. ECR/262/Inst/UP/2013/RR-19; dated 16 December 204). Written informed consent was obtained from parents or legal guardians prior to enrolment.

Study population and eligibility criteria

Neonates admitted to the NICU during the study period with biochemical evidence of conjugated hyperbilirubinemia were eligible for enrolment. Conjugated hyperbilirubinemia was defined as a direct or conjugated bilirubin level >1 mg/dL when the total serum bilirubin was ≤5 mg/dL or >20% of the total serum bilirubin when the total bilirubin was >5 mg/dL, according to standard definitions [15]. Infants whose parents or legal guardians declined participation were excluded from the study.

During the one-year study period, all consecutive admissions to the NICU who met the predefined inclusion and exclusion criteria were prospectively enrolled.

Data collection and clinical assessment

All enrolled neonates underwent a uniform, protocol‑driven diagnostic evaluation. Clinical and laboratory data were collected prospectively using a standardized case record form. Detailed maternal and perinatal information was recorded, including maternal age, parity, pregnancy-related complications (gestational diabetes mellitus, preeclampsia/eclampsia), mode of delivery, and medication exposure during pregnancy. Neonatal variables included gestational age, birth weight, admission weight, day of life at presentation, predominant feeding pattern at admission (exclusive breastfeeding, mixed feeding, or formula feeding), exposure to total or partial PN and its duration, total duration of hospital stay, history of birth asphyxia, and requirement for mechanical ventilation. Baseline laboratory investigations were performed for every infant, including complete blood count (CBC) with reticulocyte count, peripheral smear, C‑reactive protein, liver function tests (total and direct bilirubin, aspartate aminotransferase (AST), alanine aminotransferase (ALT), alkaline phosphatase (ALP)), gamma‑glutamyl transferase (GGT), serum albumin, prothrombin time (PT), INR, serum alpha‑fetoprotein (AFP), ferritin, serum lactate, thyroid function tests (TSH, T3, T4), and urine examination for reducing substances. A comprehensive infectious workup comprising blood culture, urine analysis and culture, and fungal cultures of blood and urine and abdominal ultrasonography to evaluate hepatobiliary anatomy was also performed uniformly in all neonates.

Cranial ultrasonography, blood grouping (ABO and Rh D) of both mother and infant, direct Coombs testing, cerebrospinal fluid analysis and culture, serological testing for TORCH infection and urine cytomegalovirus testing were done in cases with clinical suspicion, while tracheal aspirate cultures were obtained from ventilated neonates. In selected cases, additional targeted investigations were pursued based on clinical suspicion. These included evaluation for IEMs, hepatobiliary scintigraphy (HIDA scan), liver biopsy, urine succinylacetone estimation, galactose‑1‑phosphate uridyltransferase enzyme assay, and clinical exome sequencing.

Key variables

Sepsis

Any neonate with CRP > 6 mg/dL was considered to have sepsis.

Culture-Negative Sepsis

Infants with signs and symptoms consistent with sepsis without microbiological confirmation were categorized as those with culture-negative sepsis.

Culture-Positive Sepsis

Infants with signs and symptoms consistent with sepsis and with microbiological confirmation were categorized as those with culture-positive sepsis.

Intestinal Pathology or Intestinal Failure Associated Liver Disease (IFALD)

IFALD is hepatobiliary dysfunction caused by as a consequence of medical and surgical management strategies for intestinal failure, which can variably progress to end-stage liver disease or can be stabilized or reversed with promotion of intestinal adaptation [16].

Metabolic Disorders

Metabolic disorders are disorders resulting from enzyme deficiencies that impair metabolic pathways, leading to toxic accumulation of substrates or energy deficiency. Common metabolic disorders associated with neonatal cholestasis include galactosemia, tyrosinemia, hereditary fructose intolerance and mitochondrial hepatopathies. 

Statistical analysis

Data were analyzed using IBM SPSS Statistics for Windows, Version 24 (Released 2016; IBM Corp., Armonk, New York, United States). Continuous variables were assessed for normality and expressed as mean ± standard deviation or median with interquartile range, as appropriate. Categorical variables were presented as frequencies and percentages. Group comparisons were performed using Student’s t-test or Mann-Whitney U test for continuous variables and Chi-square or Fisher’s exact test for categorical variables. Correlation analyses were conducted using Pearson or Spearman coefficients as appropriate. A two-tailed p-value <0.05 was considered statistically significant, and 95% confidence intervals (CIs) were calculated where applicable.

Handling of missing data

Missing data were minimal (<5% for all variables). A complete case analysis approach was used, and cases with missing values for variables included in a specific analysis were excluded from that analysis. As there was a low proportion of missing data, no imputation techniques were applied.

Regression analysis and variable selection

To identify independent predictors of adverse outcomes, univariable logistic regression analysis was initially performed for all clinically relevant variables, including clinical and laboratory parameters. Statistically significant variables were then subjected to multivariable logistic regression analysis. Multicollinearity among predictor variables was assessed using the variance inflation factor (VIF), and variables with significant collinearity (VIF >5) were excluded from the final model. Adjusted odds ratios (aORs) with 95% CIs were reported.

Assessment of overfitting

**Figure 1:**
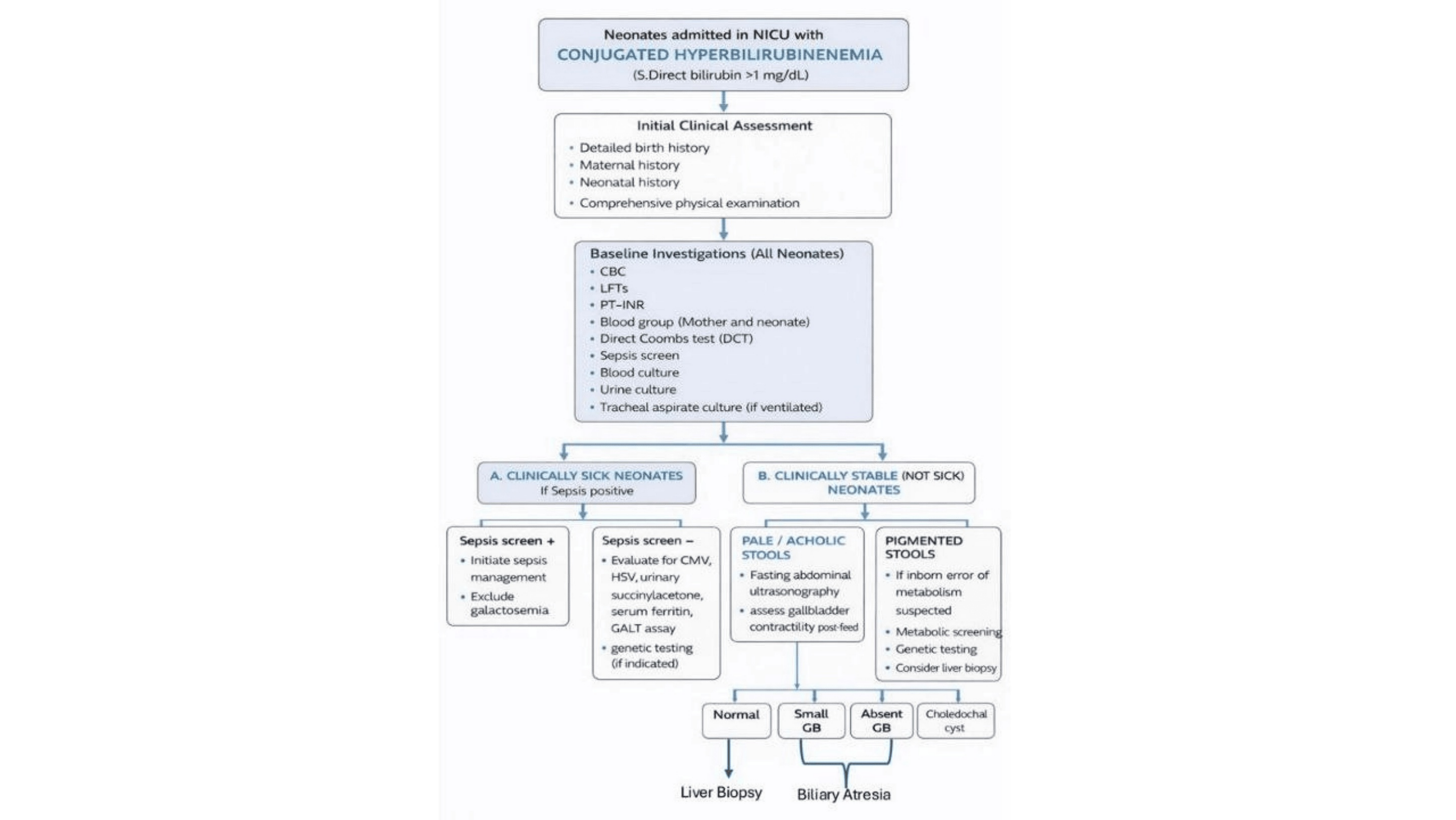
Structured Diagnostic Approach for Neonates Presenting With Conjugated Hyperbilirubinemia in the NICU, Integrating Clinical, Laboratory, and Radiological Evaluations to Guide Etiological Workup and Management NICU: Neonatal intensive care unit; CBC: Complete blood count; LFTs: Liver function tests; PT-INR: Prothrombin time-International normalized ratio; CMV: Cytomegalovirus; HSV: Herpes simplex virus; GALT: Galactose-1-phosphate uridyltransferase; GB: Gallbladder.

To minimize the risk of overfitting, the number of variables included in the multivariable model was restricted based on the principle of events per variable (EPV ≥10). Given the sample size and number of outcome events, only a limited number of predictors were retained in the final model (Figure 1). Internal validity of the model was ensured by careful variable selection and avoidance of over-parameterization.

## Results

Baseline demographic and clinical characteristics of neonates with conjugated hyperbilirubinemia

During the one-year study duration, a total of 70 neonates with conjugated hyperbilirubinemia admitted to the NICU were enrolled. There was a marked male predominance (77.1%). Although 41.4% of infants were born at term, preterm births collectively constituted 58.6% of the cohort, with 30.0% being late preterm. Low birth weight was highly prevalent, with 75.7% of neonates weighing <2500 g at birth (mean birth weight: 2.03 ± 0.63 kg), including 27.1% classified as very low birth weight. Additionally, 35.7% were small for gestational age.

Slightly more than half of the neonates were inborn (52.9%), and delivery by lower segment cesarean section was common (65.7%). The mean age of onset of jaundice was 9.37 ± 6.68 days, with over half (55.7%) presenting after the first week of life. Exclusive breastfeeding was uncommon (2.9%), while the majority required either intravenous fluids alone (40.0%) or combined fluids with enteral feeds (57.1%) during initial management (Table 1).

**Table 1:** Baseline Demographic and Clinical Characteristics of Neonates with Conjugated Hyperbilirubinemia (N = 70) SGA: Small for gestational age; AGA: Appropriate for gestational age; LGA: Large for gestational age; LSCS: Lower segment caesarean section.

Variable	Category	N	%	Mean ± SD	Test statistics	p-value
Gender	Male	54	77.1		χ² = 9.21	0.002
	Female	16	22.9			
Gestational Age	Very preterm (28–31+6 weeks)	10	14.3		χ² = 5.87	0.118
	Moderate preterm (32–33+6 weeks)	10	14.3			
	Late preterm (34–36+6 weeks)	21	30.0			
	Term (≥37 weeks)	29	41.4			
Birth Weight	Very low birth weight (<1500 g)	19	27.1		ANOVA F = 4.12	0.021
	Low birth weight (1500–2499 g)	34	48.6			
	Normal (≥2500 g)	17	24.3	2.03 ± 0.63 kg		
Size for Gestational Age	SGA	25	35.7		χ² = 12.34	0.001
	AGA	45	64.3			
	LGA	0	0			
Birth Status	Inborn	37	52.9		χ² = 0.89	0.345
	Outborn	33	47.1			
Mode of Delivery	Normal vaginal delivery	24	34.3		χ² = 6.72	0.010
	LSCS	46	65.7			
Onset of Jaundice	<24 hours	1	1.4		ANOVA F = 3.56	0.032
	1–7 days	30	42.9			
	7–14 days	23	32.9			
	>14 days	16	22.9	9.37 ± 6.68 days		
Initial Feeding Pattern	Exclusive breastfeeding	2	2.9		χ² = 15.42	
	Fluids only	28	40.0			
	Fluids + feeds (Formula/EBM)	40	57.1			

Patterns of total parenteral nutrition exposure and metabolic complications in neonates

A total of 42.9% required total parenteral nutrition (TPN), reflecting a substantial burden of feeding intolerance or gastrointestinal dysfunction within the cohort. Partial TPN was the most commonly administered modality (60%), indicating preservation of some enteral feeding capacity in the majority, whereas 20% required complete TPN, suggestive of more severe clinical compromise. Over half of the TPN recipients (53.3%) received support for 7-14 days, with a mean total duration of 9.19 ± 5.42 days, demonstrating moderate but variable exposure.

Refractory hypoglycemia requiring a glucose infusion rate >10 mg/kg/min was observed in 28.6% of cases, highlighting significant metabolic instability and possible underlying hepatic dysfunction in a substantial subset (Table 2).

**Table 2:** Total Parenteral Nutrition (TPN) Exposure and Metabolic Complications among Neonates with Conjugated Hyperbilirubinemia (N = 70) This table represents descriptive statistics. Categorical variables are expressed as frequency and percentage, while continuous variables are presented as mean ± standard deviation and range. Inferential statistical tests (Chi-square/Fisher’s exact test for categorical variables and Student’s t-test/Mann–Whitney U test for continuous variables) were applied where appropriate to assess associations with outcome variables. TPN: Total parenteral nutrition; GIR: Glucose infusion rate.

Variable	Category	N	%	Mean ± SD	Range (Min–Max)
TPN Administration	Received TPN	30	42.9		
	Did not receive TPN	40	57.1		
Type of TPN (n = 30)	Partial TPN	18	60.0	8.67 ± 3.85 days	5–22
	Complete TPN	6	20.0	10.25 ± 7.78 days	2–25
	Partial + Complete TPN	6	20.0	—	—
Duration of TPN (n = 30)	< 7 days	6	20.0		
	7–14 days	16	53.3		
	≥ 14 days	8	26.7		
	Total duration of TPN	—	—	9.19 ± 5.42 days	5–25
Refractory Hypoglycemia	Present (GIR >10 mg/kg/min)	20	28.6		
	Absent	50	71.4		

Clinical, laboratory, and microbiological profile of neonates with conjugated hyperbilirubinemia

A substantial burden of perinatal insult, multisystem involvement, and advanced hepatic dysfunction was seen in our cohort. Birth asphyxia was documented in 34.3% of neonates, with the majority exhibiting moderate to severe hypoxic-ischemic encephalopathy (HIE Stages 2 and 3), underscoring the contribution of perinatal hypoxic injury to subsequent cholestatic liver dysfunction. Clinically, all neonates presented with jaundice and dark urine, while hepatomegaly (54.3%) and splenomegaly (25.7%) were common, indicating significant hepatobiliary involvement. More than half exhibited signs of encephalopathy (52.9%), and an overwhelming proportion required oxygen support for respiratory distress (91.4%), reflecting systemic instability. Bleeding manifestations and elevated INR further suggest impaired hepatic synthetic function and coagulopathy. Ascites in 12.8% reinforces the presence of advanced liver disease in a subset. Laboratory parameters demonstrated marked cholestasis (elevated total and direct bilirubin), hepatocellular injury (raised AST and ALT), systemic inflammation (high CRP), thrombocytopenia, hypoalbuminemia, and coagulopathy. Ultrasonography findings further corroborated hepatic involvement, with hepatomegaly (60%), ascites (35.7%), and gallbladder abnormalities being frequent (Table 3).

**Table 3:** Perinatal Insults, Clinical Profile, Laboratory Parameters, and Ultrasonographic Findings in Neonates with Conjugated Hyperbilirubinemia (N = 70) Data are presented as frequency (percentage) for categorical variables and mean ± standard deviation (range) for continuous variables. Inferential statistical tests (Chi-square/Fisher’s exact test for categorical variables and Student’s t-test or Mann–Whitney U test for continuous variables) were applied where appropriate in comparative analyses. HIE: Hypoxic ischemic encephalopathy; TLC: Total leukocyte count; CRP: C-reactive protein; AST: Aspartate aminotransferase; ALT: Alanine aminotransferase; ALP: Alkaline phosphatase; INR: International normalized ratio; AFP: alpha fetoprotein; GGT: gamma-glutamyl transferase; NGRS: Non-glucose reducing substance; GALT: galactose-1-phosphate uridyltransferase; GB: Gallbladder.

Domain	Variable	Category	N	%	Mean ± SD (Range)
Birth Asphyxia	Present		24	34.3	
		HIE Stage 1	5	7.1	
		HIE Stage 2	8	11.4	
		HIE Stage 3	11	15.7	—
	Absent	—	46	65.7	—
Hemodynamic Status	Stable	—	46	65.7	—
	Unstable	—	24	34.3	—
Key Clinical Findings	Jaundice	Present	70	100	—
	Dark urine	Present	70	100	—
	Stool color	Pigmented	69	98.6	—
		Non-pigmented	1	1.4	—
	Hepatomegaly	Present	38	54.3	—
	Splenomegaly	Present	18	25.7	—
	Encephalopathy	Present	37	52.9	—
	Respiratory distress	Present	64	91.4	—
	Ascites	Present	9	12.8	—
	Bleeding manifestations	Any	46	65.7	—
Laboratory Profile	Hemoglobin (g/dL)	—	—	—	11.28 ± 3.54 (5.2–21.3)
	TLC (/mm³)	—	—	—	10,966 ± 7,041 (1180–32410)
	Platelets (lakh/mm³)	—	—	—	0.80 ± 1.16 (0.08–6.63)
	CRP (mg/dL)	—	—	—	65.17 ± 33.40 (2.42–176)
	Total bilirubin (mg/dL)	—	—	—	14.73 ± 8.31 (2.07–38.54)
	Direct bilirubin (mg/dL)	—	—	—	8.17 ± 5.44 (1.2–29.3)
	AST (U/L)	—	—	—	204.65 ± 704.21 (22.1–5950.3)
	ALT (U/L)	—	—	—	99.38 ± 183.36 (11.9–1517.8)
	ALP (U/L)	—	—	—	468.99 ± 428.57 (127.4–2844.3)
	INR	—	—	—	2.18 ± 0.87 (0.86–4.80)
	Albumin (g/dL)	—	—	—	3.12 ± 0.78 (1.56–4.56)
	AFP (ng/mL)	—	—	—	3324.9 ± 11246.9
	Ferritin (ng/mL)	—	—	—	1337.6 ± 3074.8
	GGT (U/L)	—	—	—	126.2 ± 131.1
	Blood sugar (mg/dL)	—	—	—	101.99 ± 72.58 (24–483)
Metabolic Evaluation	Lactic acidosis (>2 mmol/L)	Present	42	60.0	—
	Urine NGRS	Positive	11	15.7	—
	Hypothyroidism	Present	1	1.4	—
	Galactosemia (GALT)	Positive (2/9 tested)	2	22.2*	—
	Tyrosinemia	Positive (1/7 tested)	1	14.3*	—
	Glutathione synthase deficiency	Positive (1/6 tested)	1	16.7*	—
Ultrasonography Findings	Hepatomegaly	Present	42	60.0	—
	Splenomegaly	Present	18	25.7	—
	Ascites/fluid collection	Present	25	35.7	—
	GB edema/sludge	Present	16	22.9	—
	Heterogeneous liver echotexture	Present	5	7.1	—
	Rare findings (cyst/abscess/GB wall irregularity)	Present	1 each	1.4	—

CRP-defined sepsis (CRP >6 mg/dL) was identified in 97.1% of neonates (68/70), indicating a very high inflammatory burden in our NICU cohort. Neonates with conjugated hyperbilirubinemia represent a sick population of neonates, and as ours is a tertiary-care center, many neonates were already sick at presentation, often referred late with multiple comorbidities. Moreover, sepsis and systemic inflammation are disproportionately common in neonates with conjugated hyperbilirubinemia compared to general neonatal cohorts. Using CRP >6 mg/dL as the cut-off increases the detection rates compared to combined criteria like using CRP positivity with microbiological confirmation. Therefore, this was chosen to maximize sensitivity in a vulnerable population where missing sepsis could lead to fatal outcomes. A threshold of >6 mg/dL is considered clinically significant in many neonatal studies, reflecting systemic inflammatory response. By defining sepsis through CRP, the study aimed to show the inflammatory burden comprehensively, as culture positivity is often low in such neonates due to prior antibiotic exposure. While this threshold is sensitive for detecting systemic inflammation, it may overestimate true infection rates, particularly in neonates with hepatic dysfunction, where CRP can be elevated independent of culture-proven sepsis. Therefore, our findings should be interpreted as reflecting inflammatory burden rather than definitive microbiological confirmation of sepsis.

Culture data revealed a predominance of bacterial and fungal pathogens across infection sites. Meningitis (n=5) was exclusively bacterial, caused by Klebsiella pneumoniae (n=2) and Escherichia coli (n=3). Urosepsis (n=15) was largely fungal (Candida, n=11), with bacterial isolates including K. pneumoniae, Enterococcus faecalis, and Acinetobacter baumannii. Pneumonia confirmed by tracheal aspirate cultures (n=19) was predominantly due to Acinetobacter (n=15), followed by K. pneumoniae and Pseudomonas (n=2 each).

Bloodstream infections were documented in 66 neonates, with bacterial sepsis predominating (n=48), led by K. pneumoniae (n=25), followed by Staphylococcus (n=7), Enterococcus (n=5), Acinetobacter (n=5), and E. coli (n=3). Fungal bloodstream infections (n=13) were mainly due to Candida (n=12), while viral etiologies (n=5) included CMV and HSV.

Integrated analysis of etiology, clinical profile, and predictors of adverse outcome

This integrated analysis (Table 4) demonstrates that sepsis (41.4%) and intestinal pathology (27.1%) were the leading etiologies of neonatal conjugated hyperbilirubinemia. Mortality was substantial (50%), reflecting severe systemic illness. Adverse outcomes were significantly associated with higher bilirubin levels, coagulopathy (elevated INR), hypoalbuminemia, anemia, and especially lactic acidosis. Multivariate analysis identified neonatal acute liver failure (INR >3) as the strongest independent predictor (OR 5.4), followed by multiple organ dysfunction, refractory hypoglycemia, metabolic disease, intestinal pathology, culture-positive sepsis, and prolonged TPN exposure.

**Table 4:** Integrated Etiology, Outcomes, Laboratory Predictors, and Multivariate Risk Factors LAMA: Leave against medical advice; INR: International normalized ratio; TPN: Total parenteral nutrition; SD: Standard deviation; OR: Odds ratio; CI: Confidence interval; χ²: Chi-square.

Domain	Variable	N (%)/Mean ± SD/OR (95% CI)	Test statistics	p-value
Overall Outcomes (n=70)	Discharged	22 (31.4%)		—
	Expired	35 (50.0%)		—
	LAMA	13 (18.6%)		—
Major Etiologies	Isolated Sepsis	29 (41.4%)		—
	Intestinal pathology–related	19 (27.1%)		—
	Metabolic disorders	4 (5.7%)		—
	Biliary atresia	1 (1.4%)		—
	Congestive hepatopathy	2 (2.85%)		—
	Unidentified cause	14 (20%)		—
Significant Laboratory Differences (Good vs Bad Outcome)	Hemoglobin (g/dL)	13.83 ± 3.69 vs 10.65 ± 2.65	Student’s t-test (t ≈ 3.9)	<0.001
	Total Bilirubin (mg/dL)	11.85 ± 7.69 vs 15.44 ± 7.14	Student’s t-test (t ≈ 2.5)	0.014
	Direct Bilirubin (mg/dL)	5.79 ± 3.28 vs 8.92 ± 5.91	Mann–Whitney U (Z ≈ 2.1)	0.037
	Serum Albumin (g/dL)	3.17 ± 0.61 vs 3.00 ± 0.68	Student’s t-test (t ≈ 3.0)	0.003
	INR	1.80 ± 0.88 vs 2.31 ± 0.71	Mann–Whitney U (Z ≈ 2.1)	0.038
	Lactic acidosis (>2 mmol/L)	2 vs 40	Chi-square (χ² ≈ 15.2)	0.0001
Independent Predictors of Adverse Outcome (Multivariate Regression)	Neonatal acute liver failure (INR >3)	OR 5.40 (2.10–11.20)	Wald χ² ≈ 15.1	0.0001
	Multiple organ dysfunction	OR 4.2 (1.67–10.58)	Wald χ² ≈ 8.9	0.003
	Refractory hypoglycemia	OR 3.5 (1.41–8.68)	Wald χ² ≈ 7.5	0.006
	Metabolic cause	OR 3.1 (1.14–8.42)	Wald χ² ≈ 4.9	0.027
	Intestinal pathology	OR 2.9 (1.21–6.87)	Wald χ² ≈ 5.9	0.015
	Culture-positive sepsis	OR 2.6 (1.14–5.98)	Wald χ² ≈ 5.4	0.02
	TPN >7 days	OR 2.3 (1.01–5.12)	Wald χ² ≈ 3.9	0.047

Sepsis types, lab profiles, outcomes, and etiology

Culture-positive sepsis constituted the predominant clinical entity (77.1%) and was significantly associated with adverse outcomes (p = 0.042) (Table 5). Laboratory analysis demonstrated more severe cholestasis and coagulopathy in sepsis, particularly culture-negative cases, which showed the highest bilirubin levels and markedly prolonged INR. Thrombocytopenia and elevated inflammatory markers further characterized septic neonates. Etiologically, isolated sepsis was driven by bacterial and fungal pathogens, whereas the intestinal pathology group was defined by NEC and structural gastrointestinal disease, with no infectious overlap.

**Table 5:** Integrated Analysis of Sepsis Type, Laboratory Profile, Outcomes, and Etiological Subgroups (N = 70) TLC: Total leukocyte count; CRP: C-reactive protein; ALP: Alkaline phosphatase; PT: Prothrombin time; INR: International normalized ratio; GGT: gamma-glutamyl transferase; CMV: Cytomegalovirus; HSV: Herpes simplex virus; NEC: Necrotizing enterocolitis; TPN: Total parenteral nutrition.

Domain	Variable	Findings	Test statistics	p-value
Sepsis Distribution	No sepsis	2 (2.9%)		—
	Culture-positive sepsis	54 (77.1%)		—
	Culture-negative sepsis	14 (20.0%)		—
Key Laboratory Differences Across Sepsis Groups	TLC (cells/mm³)	Higher in culture-negative vs culture-positive (13987 ± 11192 vs 9964 ± 5339)	One-way ANOVA (F ≈ 3.4)	0.037
	Platelets	Lowest in culture-negative group (0.41 ± 0.42)	One-way ANOVA (F ≈ 4.3)	0.016
	CRP (mg/dL)	Significantly elevated in both sepsis groups	One-way ANOVA (F ≈ 3.8)	0.026
	Total Bilirubin (mg/dL)	Highest in culture-negative sepsis (17.87 ± 10.81)	One-way ANOVA (F ≈ 7.5)	0.001
	ALP (U/L)	Elevated in sepsis groups	One-way ANOVA (F ≈ 3.9)	0.023
	PT/INR	Markedly prolonged in culture-negative sepsis (INR 3.63 ± 0.54)	Kruskal–Wallis (H ≈ 12.6)	<0.001
	Serum Albumin	Lowest in culture-positive sepsis (3.01 ± 0.75)	One-way ANOVA (F ≈ 3.2)	0.042
	GGT	Significant intergroup difference	One-way ANOVA (F ≈ 3.8)	0.026
Association with Outcome	Bad outcome in culture-positive sepsis	37/54 (68.5%)	Chi-square (χ² ≈ 6.3)	0.042
	Bad outcome in culture-negative sepsis	11/14 (78.6%)		
	Bad outcome in no sepsis	0/2		
Etiological Subgroup Comparison	Isolated Sepsis (n=29)	Bacterial (18.5%), Fungal (17.1%; Candida predominant), Viral (7.1%; CMV/HSV)		—
	Intestinal Pathology (n=19)	NEC (15.7%), Intestinal atresia (7.14%), TPN cholestasis (4.28%)		—

Associations of intestinal pathology with TPN use and outcomes

In our cohort, intestinal pathology was strongly associated with TPN use (100% vs 10.3%; p = 0.004), and adverse outcomes (p = 0.010) as compared to that with the isolated sepsis group. Also, several laboratory parameters, including CRP, AST, ALT, and AFP, showed statistically significant differences between the two groups (Table 6). However, because of the small sample sizes in the subgroups (n=19 and n=29), these results should be interpreted as only hypothesis-generating and not definitive findings. Subgroup comparisons within isolated sepsis (bacterial, fungal, viral) did not demonstrate significant differences, and these analyses are underpowered. Overall, the findings highlight the strong link between intestinal pathology and TPN use, while laboratory and outcome parameter differences need further validation in larger, prospective, multicenter studies.

**Table 6:** Integrated Comparison of TPN Use, Laboratory Profile, and Outcomes Between Intestinal Pathology and Isolated Sepsis (N = 48*) *Subgroup comparison restricted to intestinal pathology and isolated sepsis cohorts. TLC: Total leukocyte count; CRP: C-reactive protein; AST: Aspartate aminotransferase; ALT: Alanine aminotransferase; ALP: Alkaline phosphatase; PT: Prothrombin time; INR: International Normalized ratio; AFP: alpha fetoprotein; GGT: gamma-glutamyl transferase

Domain	Variable	Intestinal Pathology (n=19)	Isolated Sepsis (n=29)	Test Statistics	p-value
TPN Association	TPN Present	19 (100%)	3 (10.3%)	Fisher’s Exact (χ² ≈ 28.5)	0.004
	TPN Absent	0	26 (89.7%)		
Laboratory Parameters	Hemoglobin (g/dL)	10.42 ± 3.50	13.69 ± 4.30	Student’s t-test (t ≈ 0.75)	0.458
	TLC (cells/mm³)	11114 ± 5779	10656 ± 5781	Student’s t-test (t ≈ 0.38)	0.703
	CRP (mg/dL)	66.04 ± 25.60	59.54 ± 51.61	Mann–Whitney U (Z ≈ 2.7)	0.007
	Total Bilirubin (mg/dL)	15.04 ± 9.11	14.66 ± 8.81	Student’s t-test (t ≈ 0.35)	0.722
	Direct Bilirubin (mg/dL)	8.06 ± 5.42	6.82 ± 4.98	Student’s t-test (t ≈ 0.62)	0.536
	AST (U/L)	355.08 ± 1172.07	102.59 ± 93.82	Mann–Whitney U (Z ≈ 2.8)	0.005
	ALT (U/L)	134.78 ± 298.08	60.64 ± 52.43	Mann–Whitney U (Z ≈ 2.6)	0.009
	PT/INR	24.84 ± 8.17	31.14 ± 12.57	Student’s t-test (t ≈ 2.0)	0.048
	INR	2.01 ± 0.94	2.38 ± 1.14	Student’s t-test (t ≈ 1.65)	0.104
	Serum AFP (ng/mL)	5094 ± 18014	864 ± 545	Mann–Whitney U (Z ≈ 2.1)	0.037
	GGT (U/L)	119.65 ± 123.63	135.82 ± 165.44	Student’s t-test (t ≈ 2.1)	0.041
Outcome Association	Discharged with pathology	1 (4.5%)	—	Fisher’s Exact (χ² ≈ 6.6)	0.010
	Not discharged with pathology	18 (37.5%)	—		
Isolated Sepsis Subtype vs Outcome	Bacterial (Discharge vs Not)	6 vs 6	—	Chi-square (χ² ≈ 1.5)	0.480
	Fungal (Discharge vs Not)	3 vs 9	—		
	Viral (Discharge vs Not)	2 vs 3	—		

## Discussion

This prospective NICU-based cohort of 70 neonates with conjugated hyperbilirubinemia demonstrated a distinctive etiological and prognostic pattern, characterized by a predominance of sepsis-associated cholestasis, advanced hepatic dysfunction at presentation, and high mortality (50%). In contrast to hepatology-centered or mixed referral cohorts, where obstructive and metabolic causes are more prevalent and outcomes are comparatively more favorable, the present cohort reflects the clinical severity typical of critically ill neonates [17-19].

A marked male predominance (77.1%) was observed, exceeding that reported by Sag et al. (61.1%) and Wang et al. (male: female ratio 1.8:1), whereas Yahya et al. reported a female predominance [17-19]. Such variability across geographic regions suggests that demographic differences may be influenced more by sociocultural and healthcare-related factors than by intrinsic biological susceptibility. A substantial proportion of neonates were preterm (58.6%) and had low birth weight (mean 2026 ± 634 g), making them more susceptible to cholestasis due to hepatic immaturity, systemic inflammation, intestinal pathology, and exposure to parenteral nutrition [18,20].

Clinically, dark urine was documented in all cases, underscoring its reliability as an early marker of conjugated hyperbilirubinemia. In contrast, acholic stools were infrequent (1.4%), indicating a low prevalence of extrahepatic obstructive causes in this critically ill population. Higher rates of pale stools reported by Sag et al. (41.9%) and Yahya et al. (36.1%) likely reflect a greater representation of BA and other obstructive etiologies in those cohorts [17,19]. Hepatomegaly (54.3%) and splenomegaly (25.7%) were comparable to findings by Sag et al. and Bozaci et al., supporting significant hepatobiliary involvement [17,21]. The high frequency of encephalopathy and coagulopathy further indicates advanced systemic compromise and impaired hepatic synthetic function at admission.

Biochemical findings reinforced a predominantly inflammatory intrahepatic mechanism. Total and direct bilirubin levels were higher than those reported by Yahya et al., accompanied by markedly elevated C-reactive protein and significant coagulopathy (mean INR 2.18) [19]. Compared with data from Dehghani et al., transaminase elevations were moderate, whereas alkaline phosphatase and GGT levels were lower than typically seen in obstructive cholestasis, such as BA, suggesting limited representation of mechanical obstruction and a predominance of sepsis-related hepatocellular injury [22,23]. Pronounced thrombocytopenia further reflects a severe systemic inflammatory response and septic pathology.

Sepsis was the leading etiology (41.4%), substantially exceeding the proportions reported by Sag et al. (7.6%), Wang et al. (8.7%), and Bozaci et al. (16.6%) [17,18,21]. Bloodstream infections predominated, with Gram-negative organisms and fungal pathogens contributing significantly, consistent with the high burden of nosocomial infections in tertiary NICU settings. Intestinal failure-associated liver disease (27.1%) was the second most frequent cause and was associated with markedly poor survival, highlighting the combined impact of intestinal pathology, prolonged inflammation, and nutritional compromise. Although exposure to parenteral nutrition was common, overt TPN-associated cholestasis was less frequent than reported by Wang et al. (48.8%) and Sag et al. (20.7%), possibly reflecting differences in duration of therapy, survival bias, and case mix [17,18].

Metabolic and endocrine etiologies were relatively uncommon (7.1%), compared with higher frequencies reported by Sag et al. (12.2%) [17]. Only one case of BA was identified, in contrast to higher proportions described by Sag et al. (17.6%) and Mahmud et al. (30.6%) [17,24]. This discrepancy likely reflects referral patterns, as surgically correctable conditions are frequently evaluated in specialized hepatobiliary centers rather than intensive care units. The undiagnosed rate (20%) exceeded that reported in comparable Turkish cohorts, underscoring limitations in access to advanced metabolic, genetic, and histopathological investigations in acute care environments [17,21].

Mortality (50%) was considerably higher than the rates reported by Yahya et al. (18.1%) and Wang et al. (2.5%), although it was comparable to the rate of systemic disease-associated cholestasis described by Sag et al. (44%) [17-19]. Adverse outcomes were predominantly observed among neonates with sepsis and intestinal failure-associated liver dysfunction, underscoring the role of systemic inflammation and multiorgan dysfunction as key determinants of prognosis. The severity of biochemical derangements including elevated bilirubin levels, coagulopathy, thrombocytopenia, and hypoalbuminemia further supports the association between inflammatory burden and poor outcomes in this critically ill neonatal population.

## Conclusions

In this prospective NICU cohort from a tertiary care center in Northern India, neonatal conjugated hyperbilirubinemia was predominantly attributed to sepsis (41.4%). IFALD (27.1%) was the second most common cause and was associated with poor survival. However, metabolic and obstructive causes were relatively rare. Critically ill neonates exhibited marked multisystem involvement, severe cholestasis, and an alarmingly high mortality rate (50%). This study also identified key clinical and laboratory parameters associated with adverse outcomes, including the severity of hyperbilirubinemia, coagulopathy, hypoalbuminemia, and anemia. The observed high mortality and the presence of independent predictors of adverse outcomes, such as acute liver failure, multiorgan dysfunction, refractory hypoglycemia, culture-positive sepsis, metabolic disorders, intestinal pathology, and prolonged TPN exposure, underscore the need for early recognition, prompt etiological evaluation, and aggressive multidisciplinary management. Standardized protocols for the evaluation of neonatal cholestasis, early screening for sepsis and metabolic disorders, judicious use of parenteral nutrition, and timely referral for specialized care including consideration of surgical and transplant options where indicated are essential to improve survival in these neonates. Multicenter studies incorporating comprehensive genetic and metabolic diagnostics, along with long-term follow-up, are urgently needed to refine etiological classification, guide intervention strategies, and develop predictive models for targeted neonatal care.
